# Anaphylactic Shock After First-Line Treatment With Antituberculosis Drugs in a Patient With Lupus

**DOI:** 10.7759/cureus.38862

**Published:** 2023-05-11

**Authors:** Hajar Charii, Samah Tahri, Asmae Boudouh, Hatim Kouismi, Jamal-Eddine Bourkadi

**Affiliations:** 1 Department of Pulmonology, Mohammed VI University Hospital, Oujda, MAR; 2 Faculty of Medicine and Pharmacy, Mohammed First University, Oujda, MAR; 3 Department of Internal Medicine, Mohammed VI University Hospital, Oujda, MAR; 4 Department of Pulmonology, Moulay Youssef Hospital, Ibn Sina University Hospital, Rabat, MAR

**Keywords:** antituberculosis drugs, case report, systemic lupus erythematosus, hypersensitivity reaction, anaphylaxis

## Abstract

Tuberculosis (TB) is still a major public health concern in Morocco. Although first-line antituberculosis drugs (ATD) are generally considered safe and effective, serious adverse events can occur. In this case report, we describe a female with pulmonary TB who experienced anaphylaxis induced by rifampicin (RFP) and pyrazinamide (PZA) during ATD therapy. Anaphylactic reactions to first-line ATD can occur and may lead to treatment discontinuation and challenges in finding effective alternative treatment options. Healthcare professionals should be aware of the potential of anaphylaxis with the use of these drugs, especially in patients with a history of lupus. Further research is needed to better understand the mechanisms underlying anaphylaxis and develop effective preventive and management strategies. A young female patient with a history of lupus and splenectomy presented with respiratory symptoms and deterioration of general condition. She was diagnosed with pulmonary tuberculosis and received first-line ATD, which caused complications including liver dysfunction and anaphylactic shock. Despite these challenges, the anaphylactic shock was successfully managed; she was put on a combination of levofloxacin, kanamycin, and ethambutol (ETB), as well as a desensitization protocol for isoniazid (INH); the patient was cured.

## Introduction

Anaphylaxis is defined as a severe and systemic allergic or hypersensitivity reaction that can potentially result in a life-threatening or fatal outcome [1]. In Morocco, tuberculosis (TB) is still endemic. The recommended initial course of treatment for pulmonary TB involves a combination of isoniazid (INH), rifampicin (RFP), ethambutol (ETB), and pyrazinamide (PZA), administered over a six-month period [2]. While first-line antituberculosis drug (ATD) has been demonstrated to effectively eradicate *Mycobacterium tuberculosis*, its use is still associated with a range of potential adverse side effects, such as liver dysfunction (1.74%-13.9%), gastrointestinal disturbances (1.64%-10%), and cutaneous hypersensitivity reactions (1.43%-7.5%) [2]. All first-line ATD have the potential to elicit allergic reactions [2]. Such reactions can manifest as mild symptoms, such as pruritus and erythematous eruptions, or as severe and potentially life-threatening conditions, such as anaphylactic shock [2]. In cases of significant adverse drug reactions (ADR), it is advised to discontinue the use of the incriminating drugs [3]. We present a case of an anaphylactic reaction involving first-line ATD administered to a patient following a TB diagnosis. Our case report aims to increase awareness of the clinical features of this entity and prevent fatalities.

## Case presentation

A 24-year-old female patient with a history of lupus treated with hydroxychloroquine and corticosteroids since the age of 14 and splenectomy for immune thrombocytopenia in 2012 presented to the internal medicine department with exertional dyspnea and chest pain in the context of significant weight loss, asthenia, and anorexia. Upon admission, the patient was stable, and the physical examination did not show any clinical abnormalities. During her hospitalization, a sputum smear microscopy was positive on direct examination, confirming a diagnosis of pulmonary tuberculosis. The patient immediately started a fixed-dose combination (FDC) formulation of three tablets (RFP 450 mg per day, INH 225 mg per day, PZA 1200 mg per day, and ETB 825 mg per day).

**Figure 1:**
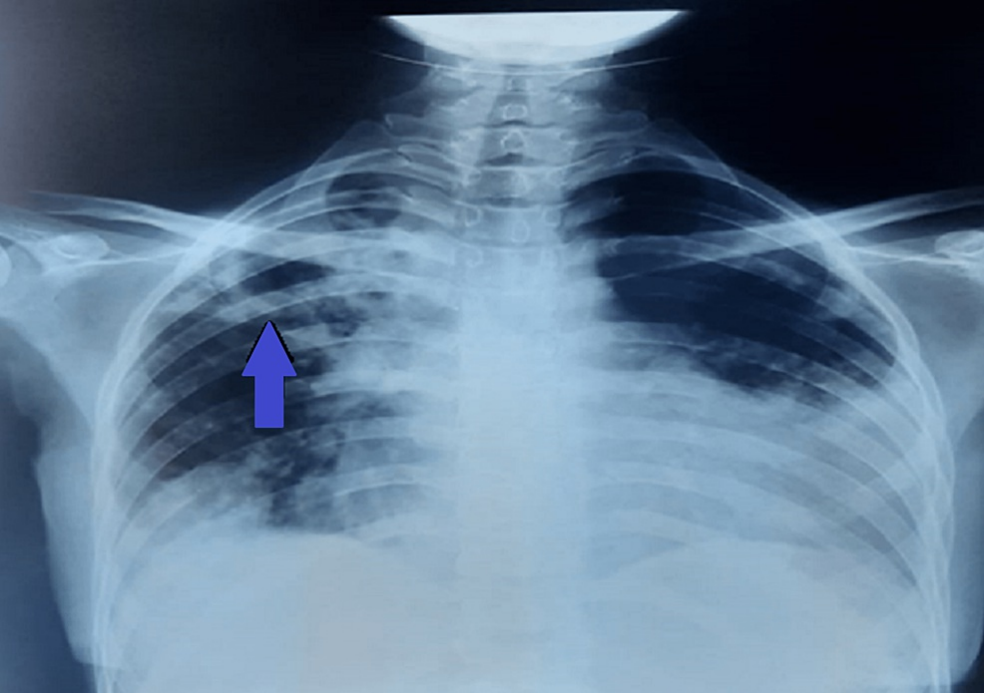
X-ray image showing bilateral reticulo-micronodular lesions and a cavitary lesion on the right upper zone (blue arrow).

On day 2, the patient developed liver dysfunction with marked elevation of transaminases (alanine aminotransferase {ALT} and aspartate aminotransferase levels four times the upper limit of normal {ULN}), and the antituberculosis treatment was stopped for 10 days until liver function tests returned to normal. On day 12, we initiated a reintroduction of antituberculosis treatment, but one hour after administration, she suffered from an anaphylactic shock that required resuscitation measures and the discontinuation of treatment. She benefited from a chest X-ray that revealed reticulo-micronodular lesions with a cavitary lesion (Figure 1).

On day 23, we started a gradual reintroduction of antituberculosis treatment: ETB (1000 mg) at full dose was well tolerated, and INH (150 mg per day) at full dose caused a fever with rash. It was stopped. PZA (1500 mg) at full dose caused hypotension, desaturation, and a skin rash, leading to its discontinuation. The decision was made to discontinue INH and PZA and to start ETB (1000 mg), levofloxacin (500 mg per day), and rifampicin (600 mg). However, the patient suffered a second anaphylactic shock, which was attributed to rifampicin (RFP).

**Figure 2:**
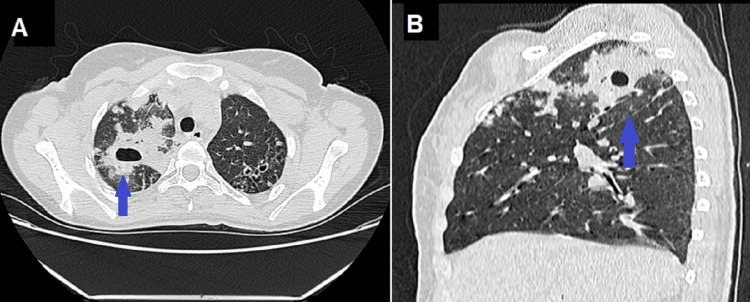
CT scan of the chest: axial (A) and sagittal (B) sections revealing a cavitary lesion with a thick wall in the left lung

Eleven months later, the patient returned with worsened clinical symptoms: cachexia with a body mass index of 15 and weight of 39 kg, dyspneic at rest and dysphonic with a performance status score of 4, bilateral basal dry crackles on lung examination, and worsening of the reticulo-micronodular lesions on CT scan (Figure 2).

Sputum smear microscopy was positive, and GeneXpert MTB/RIF (Cepheid Inc., Sunnyvale, CA) was highly positive without rifampicin resistance. The decision in a multidisciplinary meeting was to put the patient on levofloxacin 500 mg per day as a single dose, kanamycin 500 mg per day, and ethambutol 800 mg per day, introduced gradually over three days. A two-day desensitization protocol for INH (starting at a dose of 10 to the power of six) was then followed, with a daily dose of 3 mg, which was well tolerated. The patient showed clinical improvement and gained 3 kg of weight during treatment. The sputum smear microscopy was negative for acid-fast bacilli at the end of treatment; the patient was declared cured 18 months later.

## Discussion

Anaphylaxis is a serious systemic hypersensitivity reaction that often has a rapid onset and can lead to fatality. The severity of anaphylaxis is determined by compromised breathing and/or circulation, which can potentially be life-threatening; it may occur even in the absence of typical skin manifestations or circulatory shock [4]. One hypothesis regarding the evolution of immunoglobin E (IgE) in mammals suggests that these antibodies have developed to provide protection against metazoan parasites (parasitic worms and arthropods) that are too large for phagocytosis [5]. Thus, allergies could be comparable to an uncontrolled anti-parasitic response in individuals who suffer from hypersensitivity. The serum concentration of IgE in these patients can be up to 10 times higher than the normal level. In addition, allergen-specific IgE can reach a thousand times the minimum detection level found in most healthy subjects [5].

The presence of autoantibodies belonging to the IgE class has been described in various autoimmune contexts, including systemic lupus erythematosus (SLE) [5]. Recently, two studies have focused on IgE antinuclear antibody (ANA) in SLE. In the first study, Atta et al. [6] showed the presence of IgE ANA in 31.5% of SLE patients. The second study, conducted by Dema et al. [7], also showed the presence of IgE ANA in approximately 65% of patients from two SLE cohorts (French and American). Approximately 4%-5% of patients receiving ATD develop hypersensitivity reactions, necessitating the discontinuation or modification of the treatment regimen [8]. The most commonly observed hypersensitivity reactions to these drugs are cutaneous manifestations [8]. Nausea, vomiting, and abdominal pain are also reported but are especially seen in patients with severe illness secondary to drug-induced liver injury (DILI) and are reported in 50%-75% of cases [9]. Fever is noted in approximately 10% of patients, while rash is observed in 5%. Late signs of clinical worsening include overt jaundice, dark urine, and clay-colored stools. The presence of coagulopathy, hypoalbuminemia, and hypoglycemia is indicative of life-threatening hepatic dysfunction [9]. In our case, the patient denied any abdominal complaints prior to or following the marked elevation of transaminases. Although the patient tolerated ETB well at full dose after day 23, full-dose INH administration resulted in fever and rash. Similarly, full-dose PZA was followed by hypotension and dyspnea, raising concerns for anaphylaxis. Hepatotoxicity typically develops over weeks to months, in contrast to hypersensitivity reactions, which typically have a faster onset within days to weeks [9]. In our case, the transaminase elevation was observed on day 2 after treatment initiation. The regression of isoniazid-induced hepatotoxicity generally occurs over the course of several weeks. After the discontinuation of isoniazid, most patients experience a complete recovery.

According to the American Thoracic Society (ATS) guidelines, the routine monitoring of serum alanine aminotransferase (ALT) levels is not recommended except in patients with preexisting liver disorders or other risk factors for hepatotoxicity [10]. Patients are advised to discontinue INH if they experience symptoms such as nausea, abdominal pain, jaundice, or unexplained fatigue. INH should be discontinued if the serum ALT level is greater than three times the upper limit of normal (ULN) in the presence of symptoms and greater than five times the ULN in the absence of symptoms [10]. Drug reactions such as fever, rash, flu-like syndrome, hemolytic anemia, and thrombocytopenia were also linked to RFP [11]. Anaphylaxis has been nonetheless less commonly reported as a possible adverse reaction to RFP [11]. In our case, the anaphylactic shock was observed with both PZA and RFP on day 23 after the first introduction of the treatment. In our case, the multidisciplinary team's decision to initiate treatment with levofloxacin, kanamycin, and ethambutol for the patient, followed by a successful two-day desensitization protocol for INH, proved to be a highly effective course of action. The gradual introduction of the medications, along with the careful monitoring of the patient's response, resulted in a marked improvement in her clinical condition.

## Conclusions

The serious adverse events of ATD, such as life-threatening anaphylaxis, are relatively rare. However, awareness of the clinical and biological symptoms and treatment discontinuation may prevent fatal outcomes. Our case highlights the need for healthcare professionals to be aware of anaphylaxis and its clinical features, as well as its adequate management.
